# Effects of a violence prevention intervention in schools and surrounding communities: Secondary analysis of a cluster randomised-controlled trial in Uganda

**DOI:** 10.1016/j.chiabu.2018.06.007

**Published:** 2018-10

**Authors:** Katherine G. Merrill, Louise Knight, Sophie Namy, Elizabeth Allen, Dipak Naker, Karen M. Devries

**Affiliations:** a London School of Hygiene and Tropical Medicine, Department of Global Health and Development, 15-17 Tavistock Place, London, WC1H 9SH, United Kingdom; b Raising Voices, Plot 16 Tufnell Drive, Kamwokya P.O. Box 6770 Kampala, Uganda; c London School of Hygiene and Tropical Medicine, Department of Medical Statistics, Keppel Street, London, WC1E 7HT, United Kingdom

**Keywords:** Violence against children, Corporal punishment, Child health, School-Based intervention, Uganda

## Abstract

The Good School Toolkit is effective in reducing staff violence against children in Ugandan primary schools. A secondary analysis of cluster-randomised trial data was conducted to investigate intervention effects on school operational culture, and on normative beliefs and violence against children from caregivers outside of school. Students and staff completed cross-sectional surveys at baseline in 2012 and follow-up in 2014. Students’ caregivers completed follow-up surveys only. Data from 3820 students, 597 staff, and 799 caregivers were included in cross-sectional analyses at follow-up. Statistically significant intervention effects were observed for aspects of school operational culture, including students’ greater perceived emotional support from teachers and peers, students’ greater identification with their school, students’ and staffs’ lower acceptance of physical discipline practices in school, and students’ and staffs’ greater perceived involvement in school operations. Outside the school, the intervention was associated with significantly lower normative beliefs accepting the use of physical discipline practices in schools (adjusted mean difference, AMD: −0.77; 95%CI: −0.89 to −0.66; p < 0.001) and at home (AMD: −0.67; 95%CI: −0.80 to −0.54; p < 0.001), based on aggregated caregiver reports. No differences between groups were observed in past-week violence against children at home. This intervention shows promise as a platform for addressing violence against children within the school environment and surrounding community.

## 1 Introduction

Violence against children is a serious public health issue worldwide and a human rights violation, highlighted in the UN Convention on the Rights of the Child (Pinheiro, 2006). The consequences of such violence are devastating, spanning through childhood and into adulthood. Exposure to violence is a known risk factor for depression (Fergusson, Boden, & Horwood, 2008; Lansford et al., 2002; Sternberg, Lamb, Guterman, & Abbot, 2006), conduct disorder (Fergusson et al., 2008; McCabe, Hough, Yeh, Lucchini, & Hazen, 2005; Sternberg et al., 2006; Weaver, Borkowski, & Whitman, 2008), aggression (Herrenkohl, Egolf, & Herrenkohl, 1997; Lansford et al., 2002), alcohol abuse (Fergusson et al., 2008; C. Widom, Ireland, & Glyyn, 1995), poorer health status (Johnson, Cohen, Kasen, & Brook, 2002; Thomas, Hypponen, & Power, 2008), lower educational achievement (Boden, Horwood, & Fergusson, 2007; Lansford et al., 2002), and lower earnings in adulthood (CS. Widom, 1998). Beyond harming the individual child, violence against children impacts families, communities, and broader society alike by reducing children’s potential (Naker, 2017) and resulting in possible economic losses to countries (Pereznieto, Harper, Clench, & Coarasa, 2010).

Recent national surveys in sub-Saharan Africa indicate widespread reports of violence from school staff against children. More than half of young adults surveyed in Kenya (UNICEF, 2012b) and in Tanzania (UNICEF, 2011) report having experienced physical violence from a teacher before age 18. In Luwero District, Uganda, over 50% of students report having experienced physical violence and over 30% emotional violence from school staff in the past week (Devries et al., 2014). Prevalence in Uganda remains high even though corporal punishment was banned in schools by the Ugandan Ministry of Education and Sports in 1997 (UNICEF, 2012a).

School-aged children spend more time in school than in any other location besides the family home (Pinheiro, 2006), and researching violence against children in schools has been deemed a global priority (Pinheiro, 2006). Most existing research on school-based interventions has been carried out in high-income settings and has investigated intervention effects on conduct disorder (Brantley, Brantley, & Baer-Barkely, 1996; Mark Eddy, Reid, & Fetrow, 2000; Twemlow et al., 2001), bullying (Cross, Pintabona, Hall, & Hamilton, 2003; Frey et al., 2005; Stevens, De Bourdeaudhuij, & Van Oost, 2000), child sexual abuse (Dhooper & Schneider, 1995; Krahe & Knappert, 2009), and dating violence (Adler-Baeder, Kerpelman, Scramm, Higginbotham, & Paulk, 2007; Avery-Leaf, Cascardi, O’Leary, & Cano, 1997; Foshee et al., 1998) among students. School-based interventions aiming to reduce teacher violence in low or middle-income countries are almost non-existent. For example, a study on the Incredible Years programme in Jamaica found the programme to improve teachers’ positive behaviours (e.g. use of praise) and discourage negative behaviours (e.g. critical comments) (Baker-Henningham, Walker, Poweel, & Gardner, 2009), suggesting that teachers’ practices can be changed in an intervention setting.

To fill this gap in the knowledge base, the Good Schools Study was carried out from January 2012 through September 2014 in 42 primary schools in Luwero District, Uganda. The study was designed to assess the impact of the Good School Toolkit on children’s experiences of violence, incorporating four evaluation components: a cluster-randomised controlled trial (Devries, Knight, et al., 2015), a qualitative study (Kyegombe et al., 2017), an economic evaluation (Greco et al., 2016), and a process evaluation (Knight et al., 2016). The trial found the Toolkit to have resulted in a 42% reduction in risk of past-week physical violence from school staff (OR: 0.40; 95%CI: 0.26 to 0.64, p < 0.001) over an 18-month intervention period, as reported by students (Devries, Knight, et al., 2015). The trial showed improvements in students’ safety and wellbeing in school, but no changes in students’ mental health status or educational performance (Devries, Knight, et al., 2015).

### 1.1 The Good school toolkit: overview and programme theory

The Good School Toolkit is a complex school-wide intervention developed by Raising Voices, a Ugandan non-profit organisation (www.raisingvoices.org) (Devries et al., 2013). The Toolkit is designed to reduce all forms of violence in school and create a better learning environment, where students can feel safe, invest in their school, form attachments with teachers and peers, and develop a sense of belonging (Naker, 2017). Student violence and bullying victimization are less likely to occur in schools with a positive school environment, including greater perceived fairness and clarity of rules (Bradshaw, Waasdorp, Debnan, & Johnson, 2014; Gottfredson, Gottfredson, Payne, & Gottfredson, 2005), greater support from teachers (Bradshaw et al., 2014; Espelage, Polanin, & Low, 2014; Flaspohler, Elfstrom, Vanderzee, & Sink, 2009), stronger administrative commitment to a school policy on violence (Astor, Benbenishty, Vinokur, & Zeira, 2006; Bradshaw et al., 2014), stronger feelings of school attachment (Stewart, 2003), greater involvement of parents in school activities (Stewart, 2003), and intolerance of sexual harassment (Espelage et al., 2014).

Raising Voices provides schools with booklets, posters, and facilitation guides for over 60 Toolkit activities, most of which are designed to be delivered in a group setting. Activities—which include student discussions, debates, and booklet clubs—address mutual respect, power relations, non-violent discipline techniques, and classroom management strategies, among other topics. Several behaviour-change techniques are incorporated, such as setting goals, making action plans, implementing rewards and reinforcement, and creating social support for change (Abraham & Michie, 2008). Implementation takes place over 18 months through a six-step process, based on the Transtheoretical Model of Change (Prochaska & Velicer, 1997). Raising Voices staff provide a three-day training and ongoing one-on-one support to two student and two staff- ‘protagonists’ in each school. These motivated individuals are charged with engaging other staff, students, administrators, and parents with setting school-wide goals and developing action plans, with specific dates for deliverables. The protagonists facilitate activities in partnership with other school personnel. Raising Voices further supports schools to establish a *Students’ Committee*, *Teachers’ Committee*, and *Parents' Committee* which coordinate activities, and a Students’ Court designed to improve student behaviour through peer disciplining.

The Good School Toolkit centres on altering a school’s operational culture. Founded in a concept that has existed for more than 100 years (Perry, 1908), school operational culture refers to how students and staff experience, behave and feel at their school (Cohen, 2006). School ‘climate’—the term more often used in the literature—suggests prevailing conditions outside of one’s control; in contrast, ‘culture’ recognizes the reality of a school as created by its members (Naker, 2017). To fundamentally shift an environment that tolerates, incubates, and perpetuates violence, engagement is required of stakeholders at all levels within the school (teachers, administrators, students, caregivers), as well as of stakeholders outside the school (community members, siblings) (Naker, 2017). Studies show that use of violence as a form of discipline is typically a socially-normalised practice, reflecting cultures and traditions beyond the immediate school setting (Pinheiro, 2006).

**Fig. 1:**
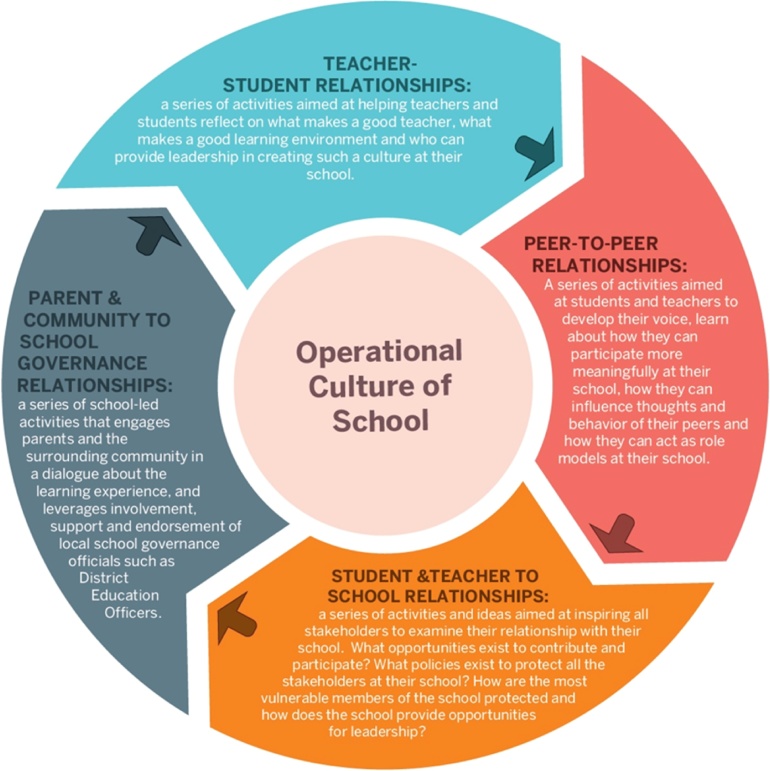
Entry-points through which the Good School Toolkit seeks to influence school operational culture.

Expanding on the work of Moos et al. (Moos, 1979), Raising Voices conceptualize school operational culture as encompassing three domains: 1) relational, referring to interpersonal relationships between teachers and students, 2) psychological, referring to attachment, belonging, identification with, and attitudes towards the school, 3) and structural, referring to policies, administrative infrastructure and capacity. To influence these domains, the Toolkit targets four entry-points: teacher-student relationships; peer-to-peer relationships, student-and-teacher-to-school relationships; and parent-and-community-to-school governance relationships (Fig. 1) (Naker, 2017). For the entry-point wheel to become animated, several additional forms of investment or influence are needed. Capacity development must occur through, for instance, the provision of trainings and materials, creation of peer learning networks, and building of violence against children prevention centres. Individuals and schools must act as legitimizers, endorsers, or amplifiers to provide leadership and model positive school operational culture for others. Policies and a legal framework must be in place—for instance, prohibiting corporal punishment in schools and facilitating the Toolkit roll-out to every school in Luwero District. Finally, local activism is needed to promote positive social norms in the community that discourage violence against children.

### 1.2 The current study

The current study presents findings from secondary analyses of the Good Schools cluster-randomised controlled trial. The study sought to assess the effects of the Toolkit on school operational culture, conceived as encompassing a relational, psychological, and structural domain. Outside of the school setting, it sought to examine differences between control and intervention groups on: normative beliefs around the acceptability of physical violence practices; and children’s experiences of physical or emotional violence from caregivers. The examination of intervention effects outside of the school was intended to shed light whether a school-wide intervention could make inroads into changing beliefs and practices in the surrounding community. Whereas the Good Schools trial addressed physical violence as its primary outcome, the current study takes a more comprehensive look at the Toolkit by also considering effects on perceptions and experiences of sexual and emotional violence. Findings will inform the development of future interventions seeking to reduce violence against children through a school-based approach.

## 2 Methods

### 2.1 Study design, population, and setting

The Good Schools Study is a two-arm cluster-randomised controlled trial with parallel assignment. A cluster design was selected given that the intervention is delivered at the school level. Clusters were comprised of primary schools in Luwero District, Uganda. A cross-sectional rather than cohort design was selected given that the trial’s main aim was to measure prevalence of violence victimization following intervention delivery. This design would also avoid problems arising from attrition of individual students, as different students were surveyed at each study time-point. Cross-sectional surveys were collected from students and school staff at baseline (in June or July 2012) and follow-up (June or July, 2014), and from primary caregivers (i.e. parents/guardians) of students at follow-up. Caregiver data were not collected at baseline, as this was not the original aim of the study. Funding was secured separately to carry out the caregiver survey as an addition to the main surveys for the trial. Luwero District, which includes both urban and rural areas, is located North of Kampala and is home to over 430,000 people. The setting was chosen as it is within the area accessible to Raising Voices.

### 2.2 Recruitment and randomization

A two-stage selection process was employed. First, schools were randomly selected. A 2010 Ministry of Education and Sports listing of all 268 primary schools registered in the Luwero District was obtained, of which 97 small schools and 20 schools with pre-existing governance interventions were excluded. The sampling frame was comprised of the remaining 151 schools, which represent 79.9% of all Primary 5, 6, and 7 students (roughly ages 11–14 years) in Luwero. Eligible schools were grouped into three strata: schools with greater than 60% girls, an approximately even ratio of boys to girls, and greater than 60% boys. Proportional to the stratum size, 42 schools were selected using a random number generator. Using stratified block randomization, 21 schools were allocated into the control and 21 into the intervention groups. It was not possible to mask participants or interviewers given the nature of the intervention. All schools agreed to participate.

Second, all individual staff members and a simple random sample of up to 130 Primary 5, 6, and 7 students were invited to participate at each school; if there were fewer than 130 Primary 5–7 students, all were invited. While implementation of the intervention was school-wide, data was collected from Primary 5–7 students only because it was thought that they would understand the survey format and procedures better than younger students. Students individually consented to participate. They were excluded if unable to speak Luganda or English, or if deemed by interviewers unable to understand the consent procedures. In line with other school-based studies in Uganda addressing sensitive topics (Twa-Twa & Oketcho, 2005), an opt-out consent process was used for students’ caregivers. Caregivers were informed of the study through information meetings held at each school and could opt out their child either in person, on the phone or in writing at several time points. Additionally, all caregivers of Primary 7 students at 38 of the 42 schools were invited to participate; data were not collected from caregivers at 4 schools because they were boarding as opposed to day schools.

### 2.3 Data collection instruments and procedures

A panel of teachers and Raising Voices staff reviewed all survey tools to ensure that items were appropriate to the local context. Tools were translated into Luganda and cognitively tested among about 40 primary school children and 20 staff in Kampala. Nearly 700 students and 40 staff from Kampala were later surveyed to explore study procedures and item distributions. The caregiver survey drew in part from the school staff survey and was piloted for length and item wording with about 25 caregivers recruited via Kampala schools.

Students, parents, and school staff were informed in advance of baseline and follow-up surveys by the head teacher. A research team—fluent in both Luganda and English and equipped with three weeks of advance training—conducted the surveys through private, individual interviews held within sight but out of earshot of others at the school. Given the low literacy level in schools, survey questions were programmed into tablet computers and read aloud to students; data collectors recorded the answers in the tablets. Students could choose to complete the interview in either Luganda or English. Interviews typically lasted 45 min. Research teams spent 3–6 days at each school collecting data; they conducted at least one repeat visit per school to identify any staff or sampled students who were absent on the day of the survey. Intervention schools received the Good School Toolkit plus implementation support throughout the study period. Control schools received no programming during the study but completed study assessments at the same time as intervention schools. After the study, control schools received the Good School Toolkit, an introductory session, and access to a peer-learning support network to support program implementation.

### 2.4 Child protection

During the informed consent process, students were informed that their details might be passed on to a child protection officer if a referral was deemed necessary. Students were also offered counselling from a trained counsellor. During survey data collection, students who had experienced abuse were referred based on predefined criteria agreed upon in advance with service providers (Child, Naker, Horton, Walakira, & Devries, 2014; Devries, Child et al., 2015a).

### 2.5 Power calculations

Given that this study is a secondary analysis of trial data, no formal power calculations were carried out. However, the main trial was powered to detect a difference of 13% in the primary outcome (past-week physical violence from school staff) with a 5% level of significance and 80% power. These calculations accounted for a potential loss-to-follow-up of up to two schools per group, a conservative estimation of 60 student interviews per school, a 50% prevalence of past-week physical violence (Devries et al., 2014), and an intracluster correlation coefficient of 0.06 (from the baseline survey) (Devries et al., 2014).

### 2.6 Outcome measures

A detailed description of outcome measures is provided in Table 1. Given the lack of existing measures of school operational culture or normative beliefs around physical discipline practices in school or at home, single and composite measures were generated for the purposes of this study. All measures used four-point Likert or Likert-type response options (DeVellis, 2012). Each response option was assigned a score of 0–3. Scores were summed and variables were modelled as continuous.

**Table 1:** Description of outcome measures.

Outcome Measure	Questionnaire items	Response Options for Each Item	Coding
**School Operational Culture**^a^			
**a) Relational**			
Student emotional support from teachers	I feel that my teachers care about me.	•All the time •Most of the time •Sometimes •Never	Score range 0 (low) to 3 (high).
Student emotional support from peers	(a) I have friends that I can talk to about important things; (b) I have friends that I can count on for support.	•All the time •Most of the time •Sometimes •Never	Score range 0 (low) to 6 (high).
Staff perceived relationship with students	(a) Would you say that students feel comfortable talking with you/want to confide in you if they are unhappy about something at home or at school? (b) Do you feel that students respect their peers and adults? (c) Do school staff respect their students? (d) Do you have a good relationship with the students?	•All the time •Most of the time •Sometimes •Never	Score range 0 (low) to 12 (high).
Staff perceived relationship with colleagues	(a) Do you feel there is anybody at your school you can talk to if you are unhappy about work? (b) Thinking about your school as a whole, do you feel like you are part of a team?	•All the time •Most of the time •Sometimes •Never	Score range 0 (low) to 6 (high).
Staff perceived relationship with caregivers	Do you have a good relationship with parents?	•All the time •Most of the time •Sometimes •Never	Score range 0 (low) to 3 (high).
Caregiver perceived relationship with staff	Do you have a good relationship with school staff?	•All the time •Most of the time •Sometimes •Never	Score range 0 (low) to 3 (high).
**b) Psychological**			
Student identification with school	(a) I feel safe in school; (b) I feel like I belong in school; (c) I like to spend time at school.	•All the time •Most of the time •Sometimes •Never	Score range 0 (low) to 9 (high).
Staff identification with school	(a) How often would you say that you enjoy your job? (b) Do you feel valued as an employee? (c) Do you feel that your employers care about your wellbeing?	•All the time •Most of the time •Sometimes •Never	Score range 0 (low) to 9 (high).
Student acceptance of physical discipline in school	(a) Teachers must hit students to make them listen; (b) Students should fear their teachers; (c) Teachers must hit students to make them learn.	•All the time •Most of the time •Sometimes •Never	Score range 0 (low) to 9 (high).
Staff acceptance of physical discipline in school	(a) Physical discipline of students by teachers is normal; (b) Sometimes teachers must hit students to make them listen; (c) Students who misbehave should be physically disciplined; (d) Sometimes teachers must hit students to make them learn; (e) Sometimes physically disciplining students is the only way to make them respect you; (f) It is OK to physically discipline children when they misbehave.	•Strongly agree •Agree •Disagree •Strongly disagree	Scores range 0 (low) to 18 (high).
Student acceptance of sexual violence from teachers in school	It is OK for a teacher to have sex with a female student if she gives consent.	•Strongly agree •Agree •Disagree •Strongly disagree	Score range 0 (low) to 3 (high).
**c) Structural**			
Student perceived involvement in school operations	In your school, are students' views about how to improve the school taken seriously by adults who work at the school?	•All the time •Most of the time •Sometimes •Never	Score range 0 (low) to 3 (high).
Staff perceived involvement of *staff* in school operations	(a) In your opinion, do you have enough opportunities to say what you think and contribute to how the school is run? (b) Do you feel that your views on how the school's policies could be improved are welcomed?	•All the time •Most of the time •Sometimes •Never	Scores range 0 (low) to 6 (high).
Staff perceived involvement of *students* in school operations	(a) Do students in your school have an opportunity to say what they think? (b) Do students in your school have an opportunity to contribute to how the school is run?	•All the time •Most of the time •Sometimes •Never	Scores range 0 (low) to 6 (high).
Caregiver perceived involvement in school operations	(a) Do you have enough opportunities to say what you think and contribute to how the school is run? (b) Do you feel that your views on how the school's policies could be improved are welcomed?	•All the time •Most of the time •Sometimes •Never	Scores range 0 (low) to 6 (high).
**Normative Beliefs**^a^			
Acceptability of physical discipline in school	(a) Sometimes teachers must hit students to make them listen; (b) Students should fear their teachers; (c) Students who misbehave should be physically disciplined; (d) Sometimes teachers must hit students to make them learn.	•Strongly agree •Agree •Disagree •Strongly disagree	Score range 0 (low) to 12 (high). Mean scores by school calculated.
Acceptability of physical discipline at home	(a) Sometimes parents must hit children to make them listen; (b) Children should fear their parents; (c) Children who misbehave should be physically disciplined; (d) Sometimes parents must hit children to make them learn.	•Strongly agree •Agree •Disagree •Strongly disagree	Scores range 0 (low) to 12 (high). Mean scores by school calculated.
Acceptability of sexual violence from teachers at school	It is OK for a teacher to have sex with a female student if she gives consent.	•Strongly agree •Agree •Disagree •Strongly disagree	Score range 0 (low) to 3 (high).
**Violence against Children at Home**^b^			
Past-week experience of physical violence from a parent or caregiver (child reports)	Has a parent/caregiver [done the following in the past week]: (a) Twisted your arm or any other body part, slapped you, pushed you, or thrown something at you? (b) Punched you, kicked you, or hit you with a closed fist? (c) Hit you with an object, such as a stick or a cane, or whipped you? (d) Cut you with a sharp object or burnt you?	•Yes •No	Coded 1 if “Yes” to any of the items; coded 0 if “No” to all items.
Past-week experience of emotional violence from a parent or caregiver (child reports)	Has a parent/caregiver [done the following in the past week]: (a) Insulted you, or called you rude or hurtful names? (b) Accused you of witchcraft? (c) Locked you out or made you stay outside? (d) Not given you food?	•Yes •No	Coded 1 if “Yes” to any of the items; coded 0 if “No” to all items.
Past-week use of physical violence against child (caregiver reports)	Have you done the following things to your child in response to misbehaviour or at any other time [in the past week]: (a) Twisted their arm or any other body part, slapped them, pushed them, or thrown something at them? (b) Punched them, kicked them, or hit them with a closed fist? (c) Hit them with an object, such as a stick or a cane, or whipped them? (d) Cut them with a sharp object or burnt them?	•Yes •No	Coded 1 if “Yes” to any of the items; coded 0 if “No” to all items.
Past-week use of emotional violence against child (caregiver reports)	Have you done the following things to your child in response to misbehaviour or at any other time [in the past week]: (a) Insulted them, or called them rude or hurtful names? (b) Accused them of witchcraft? (c) Locked them out or made them stay outside? (d) Not given them food?	•Yes •No	Coded 1 if “Yes” to any of the items; coded 0 if “No” to all items.

a Response options for each item coded as 0-3. Scores summed, modelled as continuous variables.

b Adapted from the IPSCAN Child Abuse Screening Tool (ICAST) and the WHO Multi-Country Study on Women’s Health and Domestic Violence against Women.

School operational culture was assessed by investigating relational, psychological, and structural domains. The relational domain examined: students’ feelings of emotional support from teachers (1 item) and peers (2 items); staffs’ perceived relationship with students (4 items), colleagues (2 items), and caregivers (1 item); and caregivers’ perceived relationship with staff (1 item). The psychological domain assessed: degree of identification with the school among students (3 items) and staff (3 items); acceptance of physical discipline practices in school among students (3 items) and staff (6 items); and acceptance of sexual violence from teachers among students (1 item). The structural domain examined: students’ perceived level of involvement with school operations (1 item); staffs’ perceived level of involvement in school operations among staff (2 items) and students (2 items); and caregivers’ perceived level of involvement in school operations (2 items).

To investigate the effects of the intervention outside of the school setting, caregiver attitudes were analysed as a proxy measure for normative beliefs regarding violence against children. Composite variables were generated to assess caregivers’ perceived acceptability of physical discipline practices in school (4 items) and at home (4 items) using caregiver reports. A single item assessed caregivers’ perceived acceptability of sexual violence from teachers. Scores were aggregated by school to approximate approval or disapproval of physical disciplinary practices within communities surrounding schools.

Finally, acts of violence against children taking place in the home were assessed. Children’s reported experience of physical or emotional violence from a parent or caregiver in the past week, as well as caregivers’ self-reported use of physical or emotional violence against their child in the past week were modelled. Experiences of violence were measured using questions about behaviourally-specific acts of violence adapted from the standardized IPSCAN Child Abuse Screening Tool (ICAST) (IPSCAN, 2006) and the WHO Multi-Country Study on Women’s Health and Domestic Violence against Women (WHO MCS) (Table 1) (Garcia-Moreno, Ellsberg, Heise, & Watts, 2005). The ICAST was generated by over 60 scholars from developing and developed countries and has been administered in numerous sub-Saharan African settings (IPSCAN, 2006). The WHO MCS has been widely-employed and validated among males and females in ranging settings internationally (Fulu, Jewkes, Roselli, & Garcia-Moreno, 2013; Nybergh, Taft, & Krantz, 2013; Schraiber, Latorre, Franca, Segri, & D'Oliveira, 2010).

### 2.7 Statistical analysis

Analyses centred on a cross-sectional comparison of follow-up data between control and intervention groups, using the principle of intention-to-treat. Data comprised a two-level hierarchical structure. Students and staff were clustered by school (i.e. each student and staff member was associated with only one school). Students’ caregivers at a given school were conceived as forming part of a ‘community’ for that particular school; each caregiver was associated with only one school, and caregiver reports were aggregated by school to approximate normative beliefs in communities surrounding schools. Hence, analyses used mixed-effects regression models with a random effect at the school level to account for clustering of students, staff, and caregivers within schools.

All outcomes for school operational culture and normative beliefs were modelled as continuous. Mean differences between control and intervention schools at follow-up, along with associated 95% confidence intervals and p values, were estimated using mixed-effects linear regression. To account for some observed cases of non-normally distributed variables, 2000 bootstrap replications were used. Basic models for continuous outcomes included as a covariate the school-level mean of the outcome at baseline, except in the case of caregiver measures given a lack of baseline data. Adjusted models additionally controlled for the school’s baseline level of past-week physical violence from school staff (modelled as a continuous variable at the school level) and the school’s location (urban or rural). Adjusted individual-level models controlled for the individual’s sex and, in the case of students, disability status.

All outcomes for violence against children in the home were modelled as binary. Odds ratios comparing intervention to control schools at follow-up, 95% confidence intervals (CIs) and p values were estimated using mixed-effects logistic regression. Basic models for binary outcomes made no adjustment for baseline characteristics. Adjusted models controlled for the school’s baseline level of past-week physical violence from school staff, the school’s location (urban or rural), the individual’s sex, and, in the case of students, disability status. Analyses were performed using STATA 14.

### 2.8 Ethics

The trial is registered at clinicaltrials.gov (NCT01678846). Ethical approval for the trial was obtained from the London School of Hygiene and Tropical Medicine (Reference: 6183) and the Uganda National Council of Science and Technology (Reference: SS 2520).

## 3 Results

**Fig. 2:**
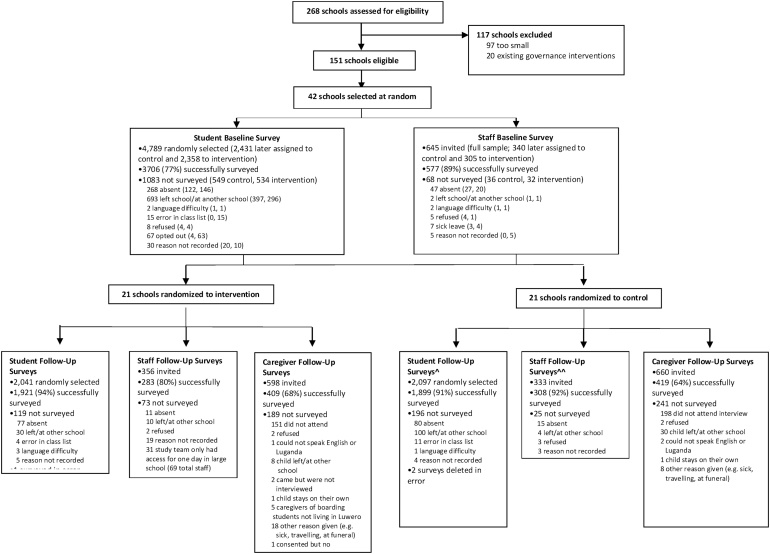
Flow chart showing trial profile. ^In a previous publication (Devries, Child, Elbourne, Naker, & Heise, 2015), we inadvertently omitted mention of 2 student follow-up surveys in the control group which were erroneously deleted. ^^In a previous publication (Kayiwa et al., 2017), we inadvertently counted 329 eligible staff in the control group at follow-up when we should have counted 333.

Surveys were administered in 42 schools, 21 control and 21 intervention. Cross-sectional surveys were completed by 3706 students at baseline (77%) and 3820 at follow-up (92%). Of staff invited, 89% (n = 577) completed surveys at baseline and 86% (n = 591) at follow-up. Of caregivers invited at follow-up, 66% (n = 828) completed surveys. Data from 29 caregivers were excluded given missing data on the caregiver’s sex, resulting in 799 surveys analysed (n = 403 control and 396 intervention). Fig. 2 presents the flow of participants through the trial.

### 3.1 Characteristics of schools, students, staff, and caregivers

About one third of schools were urban (36%) (Table 2). On average, over 53% of students across schools reported experiencing physical violence from school staff in the past week. The mean age of students was 13 years (SD: 1.5), most ranging in age from 11 to 14. Just over half were female and reported eating less than three meals a day. Over 7% had some form of disability. The average age for staff was nearly 35 years (SD: 8.6), while caregivers were generally in their early forties (mean age: 42.3, SD: 12.0). For staff and caregivers alike, more than half the sample was female, about two-thirds were Baganda, and a majority were either Roman Catholic or Anglican. About a third of staff reported owning their housing property, compared with over 80% of caregivers.

**Table 2:** Characteristics of schools, students, staff, and caregivers^a^.

		Control	Intervention	All Schools
**School Characteristics**				
Total sample (n)		21	21	42
Urban location		8 (38.1%)	7 (33.3%)	15 (35.7%)
Prevalence of past-week physical violence, mean (SD)*		54.3 (11.7)	52.8 (13.1)	53.6 (12.3)
**Student Characteristics**				
Total sample (n)		1182	1824	3706
Age (years), mean (SD)		13.0 (1.5)	13.1 (1.5)	13.0 (1.5)
Female sex		1,010 (53.7%)	927 (50.8%)	1937 (52.3%)
School class				
	5	703 (37.4%)	739 (40.5%)	1442 (38.9%)
	6	697 (37.0%)	644 (35.3%)	1341 (36.2%)
	7	482 (25.6%)	441 (24.2%)	923 (24.9%)
Some disability		142 (7.5%)	129 (7.1%)	271 (7.3%)
Meals eaten in previous day				
	1 meal	250 (13.3%)	266 (14.6%)	516 (13.9%)
	2 meals	743 (39.5%)	700 (38.4%)	1443 (38.9%)
	3+ meals	888 (47.2%)	858 (47.0%)	1746 (47.1%)
**Staff Characteristics**				
Total sample (n)		304	273	577
Age (years), mean (SD)		35.1 (8.7)	33.8 (8.3)	34.5 (8.6)
Female sex		177 (58.2%)	161 (59.0%)	338 (58.6%)
Baganda tribe		196 (64.5%)	166 (60.8%)	362 (62.7%)
Religion				
	Roman Catholic	87 (28.7%)	74 (27.3%)	161 (28.0%)
	Anglican	94 (31.0%)	103 (38.0%)	197 (34.3%)
	Pentecostal	54 (17.8%)	42 (15.5%)	96 (16.7%)
	Muslim	34 (11.2%)	37 (13.7%)	71 (12.4%)
	Seventh Day Adventist	34 (11.2%)	15 (5.5%)	49 (8.5%)
Housing status				
	Owns	112 (36.8%)	87 (32.0%)	199 (34.5%)
	Rents	91 (29.9%)	73 (26.8%)	164 (28.5%)
	Employer pays	74 (24.3%)	95 (34.9%)	169 (29.3%)
	Lives somewhere without paying	25 (8.2%)	15 (5.5%)	40 (6.9%)
	Other	2 (0.7%)	2 (0.7%)	4 (0.7%)
**Caregiver Characteristics**				
Total sample (n)		403	396	799
Age (years), mean (SD)		42.4 (11.3)	42.4 (12.5)	42.4 (11.9)
Female sex		237 (58.8%)	210 (53.0%)	447 (55.9%)
Baganda tribe		276 (68.5%)	248 (62.6%)	524 (65.6%)
Religion				
	Roman Catholic	120 (29.8%)	120 (30.2%)	240 (30.0%)
	Anglican	156 (38.7%)	143 (36.0%)	299 (37.4%)
	Pentecostal	44 (10.9%)	29 (7.3%)	73 (9.1%)
	Muslim	65 (16.1%)	89 (22.5%)	154 (19.3%)
	Seventh Day Adventist	15 (3.7%)	14 (3.5%)	29 (3.6%)
Housing status				
	Owns	332 (82.4%)	329 (83.1%)	661 (82.7%)
	Rents	39 (9.7%)	36 (9.1%)	75 (9.4%)
	Employer pays	5 (1.2%)	9 (2.3%)	14 (1.8%)
	Lives somewhere without paying	27 (6.7%)	22 (5.6%)	49 (6.1%)

a Characteristics of schools, students, and staff reported at baseline; characteristics of caregivers reported at follow-up, given that no data was collected from caregivers at baseline. Data on religion were not collected from students. *The mean per school refers to the average proportion of students reporting physical violence from school staff in the past week.

### 3.2 Outcomes at baseline

Outcomes were generally evenly distributed across study groups at baseline (Table 3). Student acceptance of sexual violence from teachers in school was low, as were student reports of past-week physical or emotional violence from a parent or caregiver.

**Table 3:** Outcomes at baseline among students and staff^a^.

	Control	Intervention	All Schools
Students	1,882	1,824	3706
Staff	304	273	577
**School Operational Culture**	**Mean(SD)**		
**a) Relational**			
Student emotional support from teachers (0-low to 3-high)	2.25 (0.88)	2.24 (0.88)	2.25 (0.88)
Student emotional support from peers (0-low to 6-high)	3.15 (1.7)	3.04 (1.7)	3.10 (1.73)
Staff perceived relationship with students (0-low to 12-high)	8.02 (2.09)	8.29 (1.93)	8.15 (2.02)
Staff perceived relationship with colleagues (0-low to 6-high)	4.71 (1.26)	4.60 (1.36)	4.66 (1.31)
Staff perceived relationship with caregivers (0-low to 3-high)	2.15 (0.81)	2.17 (0.84)	2.16 (0.82)
**b) Psychological**			
Student identification with school (0-low to 9-high)	7.08 (1.76)	7.02 (1.75)	7.05 (1.75)
Staff identification with school (0-low to 9-high)	5.28 (2.13)	5.63 (2.06)	5.45 (2.10)
Student acceptance of physical discipline practices in school (0-low to 9-high)	5.05 (2.5)	4.72 (2.6)	4.88 (2.55)
Staff acceptance of physical discipline practices in school (0-low to 18-high)	7.49 (3.21)	6.67 (3.55)	7.10 (3.40)
Student acceptance of sexual violence from teachers (0-low to 3-high)	0.11 (0.43)	0.11 (0.43)	0.11 (0.43)
**c) Structural**			
Student perceived involvement in school operations (0-low to 3-high)	1.85 (1.00)	1.83 (1.02)	1.84 (1.01)
Staff perceived involvement of staff in school operations (0-low to 6-high)	3.22 (1.50)	3.35 (1.63)	3.28 (1.57)
Staff perceived involvement of students in school operations (0-low to 6-high)	2.83 (1.54)	2.93 (1.66)	2.87 (1.59)
**Violence against Children in the Home**	**n(%)**		
Past-week experience of physical violence from a parent or caregiver (reported by child)	48 (2.6%)	51 (2.8%)	99 (2.67%)
Past-week experience of emotional violence from a parent or caregiver (reported by child)	42 (2.2%)	29 (1.6%)	71 (1.92%)

a Data from students’ caregivers not collected at baseline.

### 3.3 Intervention effects

Comparison of basic and adjusted models (Table 4) showed little confounding by the school’s baseline level of past-week physical violence from school staff, the school’s location (urban/rural), the individual’s sex, or the student’s disability status (for student models).

**Table 4:** Intervention effects on school operational culture, normative beliefs, and violence against children in the home.

	Summary Statistics		Intervention Effect			
	**Control**	**Intervention**	**Basic Model**	**p value**	**Adjusted Model**	**p value**
Students	1,899	1,921				
Staff	308	283				
Caregivers	403	396				
**School Operational Culture**	**Mean (SD)**		**Mean Difference (95%CI)**			
**a) Relational**						
Student emotional support from teachers (0-low to 3-high)	2.30 (0.84)	2.41 (0.80)	0.10 (0.05 to 0.16)	<0.001	0.10 (0.04 to 0.16)	0.002
Student emotional support from peers (0-low to 6-high)	3.26 (1.8)	3.47 (1.8)	0.26 (0.08 to 0.45)	0.005	0.25 (0.06 to 0.43)	0.007
Staff perceived relationship with students (0-low to 12-high)	8.14 (2.02)	8.48 (2.05)	0.29 (−0.74 to 0.65)	0.12	0.33 (−0.06 to 0.71)	0.09
Staff perceived relationship with colleagues (0-low to 6-high)	4.36 (1.35)	4.55 (1.31)	0.15 (−0.89 to 0.40)	0.22	0.13 (−0.14 to 0.40)	0.34
Staff perceived relationship with caregivers (0-low to 3-high)	2.15 (0.82)	2.24 (0.78)	0.09 (−0.04 to 0.21)	0.19	0.10 (−0.03 to 0.23)	0.14
Caregiver perceived relationship with staff (0-low to 3-high)	2.28 (0.81)	2.38 (0.74)	0.13 (−0.02 to 0.27)	0.08	0.13 (−0.02 to 0.28)	0.10
**b) Psychological**						
Student identification with school (0-low to 9-high)	7.30 (1.74)	7.50 (1.66)	0.24 (0.09 to 0.40)	0.002	0.23 (0.07 to 0.40)	0.005
Staff identification with school (0-low to 9-high)	5.60 (2.07)	5.63 (1.92)	0.05 (−0.25 to 0.35)	0.75	0.11 (−0.21 to 0.44)	0.54
Student acceptance of physical discipline practices in school (0-low to 9-high)	5.29 (2.4)	3.85 (2.7)	−1.46 (−1.92 to −1.00)	<0.001	−1.51 (−1.95 to −1.07)	<0.001
Staff acceptance of physical discipline practices in school (0-low to 18-high)	7.06 (3.14)	4.46 (2.95)	−2.59 (−3.27 to −1.92)	<0.001	−2.49 (−3.15 to −1.84)	<0.001
Student acceptance of sexual violence from teachers (0-low to 3-high)	0.07 (0.35)	0.07 (0.35)	−0.002 (−0.04 to 0.03)	0.90	−0.004 (−0.04 to 0.03)	0.80
**c) Structural**						
Student perceived involvement in school operations (0-low to 3-high)	1.95 (0.99)	2.23 (0.88)	0.30 (0.21 to 0.39)	<0.001	0.28 (0.19 to 0.37)	<0.001
Staff perceived involvement of staff in school operations (0-low to 6-high)	3.30 (1.62)	3.73 (1.57)	0.42 (0.17 to 0.68)	0.001	0.42 (0.17 to 0.68)	<0.001
Staff perceived involvement of students in school operations (0-low to 6-high)	2.72 (1.53)	3.85 (1.45)	1.20 (0.86 to 1.55)	<0.001	1.20 (0.84 to 1.57)	<0.001
Caregiver perceived involvement in school operations (0-low to 6-high)	3.16 (1.8)	3.33 (1.8)	0.21 (−0.11 to 0.54)	0.20	0.21 (−0.21 to 0.64)	0.32
**Normative Beliefs**^a^	**Mean (SD)**		**Mean Difference (95%CI)**			
Acceptability of physical discipline practices in school (0-low to 12-high)	6.95 (0.74)	6.19 (0.93)	−0.76 (−.88 to −0.64)	<0.001	−0.77 (−0.89 to −0.66)	<0.001
Acceptability of physical discipline practices at home (0-low to 12-high)	6.62 (0.93)	5.96 (0.92)	−0.66 (−0.79 to −0.53)	<0.001	−0.67 (−0.80 to −0.54)	<0.001
Acceptability of sexual violence from teachers at school (0-low to 3-high)	0.48 (0.17)	0.49 (0.18)	0.01 (−0.02 to 0.03)	0.61	0.01 (−0.02 to 0.03)	0.51
**Violence against Children in the Home**	**n(%)**		**Odds Ratio (95%CI)**			
Past-week experience of physical violence from a parent or caregiver (reported by child)	36 (1.8%)	32 (1.6%)	0.87 (0.44–1.70)	0.68	0.82 (0.44–1.54)	0.54
Past-week experience of emotional violence from a parent or caregiver (reported by child)	23 (1.2%)	23 (1.2%)	0.98 (0.55–1.76)	0.95	0.97 (0.54–1.74)	0.92
Past-week use of physical violence against child (reported by caregiver)	20 (5.0%)	16 (4.0%)	0.79 (0.39–1.62)	0.52	0.79 (0.39–1.59)	0.51
Past-week use of emotional violence against child (reported by caregiver)	49 (12.2%)	43 (10.8%)	0.88 (0.57–1.36)	0.56	0.88 (0.57–1.36)	0.56

Notes: Mean difference = mean outcome in intervention – mean outcome in control. In logistic models, “intervention” is coded as 1.0. Mixed-effects regression models were used to account for clustering within schools. Missing data was very low in quantity (2.5% or less, with most variables showing less than 0.1% missing data) and similarly distributed across study arms. Mean differences with associated 95%CIs were calculated for continuous outcomes (i.e. school operational culture and normative beliefs) and odds ratios with associated 95% CIs were calculated for binary outcomes (i.e. violence against children in the home) at follow-up. The basic model for continuous outcomes adjusted for the school-level mean of the outcome at baseline, except in the case of caregiver measures given a lack of baseline data. Adjusted models for continuous outcomes additionally controlled for the school’s baseline level of past-week physical violence from school staff (modelled as a continuous variable at the school level) and the school’s location (urban or rural). Adjusted individual-level models controlled for the individual’s sex and, in the case of students, disability status. The basic model for binary outcomes made no adjustment for baseline levels. Adjusted models for binary outcomes controlled for the school’s baseline level of past-week physical violence from school staff, the school’s location (urban or rural), the individual’s sex, and, in the case of students, disability status. For non-normally distributed continuous outcomes, 95%CIs were estimated using 2000 bootstrap replications.

a Measures represent mean scores for aggregated caregiver reports.

In the relational domain of school operational culture (Table 4), students from intervention schools reported greater feelings of emotional support from teachers than did students in control schools (adjusted mean difference, AMD: 0.10, 95%CI: 0.04–0.16, p < 0.01). Strong evidence was also observed for students’ feelings of emotional support from peers (AMD: 0.25, 95%CI: 0.06–0.43, p < 0.01). No other effects were observed for the remaining relational domain factors. Within the psychological domain, very strong evidence was observed for lower acceptance of physical discipline practices in intervention compared with control schools among students (AMD: −1.51; 95%CI: −1.95 to −1.07, p < 0.001) and staff (AMD: −2.49; 95%CI: −3.15 to −1.84, p < 0.001). Students also reported higher levels of identification with their school in the intervention compared with control group (AMD: 0.23, 95%CI: 0.07–0.40, p < 0.01). No such effects were observed among staff. No evidence was observed for students’ acceptance of sexual violence from teachers. Very strong evidence of an intervention effect was observed for the structural domain of school operational culture. Students were more likely to report feeling involved in school operations in intervention versus control groups (AMD: 0.28, 95%CI: 0.19–0.37, p < 0.001). Staff also reported perceiving a greater opportunity for involvement in school operations for staff themselves (AMD: 0.42, 95%CI: 0.17–0.68, p < 0.001) and for students (AMD: 1.20, 95%CI: 0.84–1.57, p < 0.001) in intervention compared with control groups.

Significant differences between control and intervention groups were observed in normative beliefs regarding the acceptability of physical discipline practices among caregivers (p < 0.001). At follow-up, acceptability of physical discipline practices in school was significantly lower in communities surrounding intervention compared with control schools (AMD: −0.77; 95%CI: −0.89 to −0.66). Communities surrounding intervention schools were also less likely to report acceptance of physical discipline practices in the home setting when compared to control communities (AMD: −0.67; 95%CI: −0.80 to −0.54). No evidence was observed for differences between groups on normative beliefs around teachers’ use of sexual violence against students. Additionally, no significant differences were observed for measures of violence against children in the home in the past week, although all effect estimates were in the direction of lower reported levels of violence (i.e. AMD in the expected direction) in intervention schools.

## 4 Discussion

Based on the findings of this secondary trial analysis, the Good School Toolkit produced significant effects on aspects of the relational, psychological, and structural domains of school operational culture in primary schools in Luwero District, Uganda. Importantly, intervention effects were also observed beyond the school setting; there was very strong evidence of a difference between control and intervention schools in normative beliefs around the acceptability of physical discipline practices at school and at home, based on aggregated reports from students’ caregivers at follow-up. These findings suggest that school-based interventions show promise in producing positive changes not only within the school setting but also in surrounding communities.

Positive changes were observed for many aspects of school operational culture at follow-up, suggesting that the Toolkit is effective in improving the school learning environment. Changes spanning the relational, psychological, and structural domains are likely the result of intentional activities delivered through the Toolkit. The intervention facilitated student discussions, debates, booklet clubs, and other activities, which may have increased students’ feelings of emotional support from peers and sense of identification with their school. The intervention also emphasized participation in school operations through the creation of student and staff governance committees and student courts. These activities could explain changes in the perceived involvement of students and staff in school operations. While the Toolkit intends to engage caregivers through Parent Committees, this engagement has typically extended to only a few caregivers per school. Monitoring records for the Good School Study indicate that these Parent Committees were not fully functional in all schools. The inconsistent parent/caregiver engagement may explain why caregivers’ relationships with staff and perceived involvement in school operations did not improve in intervention schools. Additionally, no intervention effects were observed for either students’ or caregivers’ acceptance of sexual violence from teachers. This may be because the acceptance of sexual violence measures were in each case based on only one item.

Findings on changes in school operational culture are consistent with the main trial outcome regarding observed reductions in staff members’ use of physical violence (Devries, Knight, et al., 2015). As staff in intervention schools shifted away from physical discipline practices, it makes sense that students reported a corresponding increase in levels of emotional support received from teachers. In the psychological domain, the large reductions observed in staff’s acceptance of physical discipline practices is also consistent with the main trial outcome. The literature demonstrates associations between attitudes towards physical discipline and use of such forms of discipline by teachers (Khoury-Kassabri, 2009; Khoury-Kassabri, Attar-Schwartz, & Zur, 2013) and parents alike (Ateah & Durrant, 2005; Vittrup, Holden, & Buck, 2006). Such associations are supported by the Theory of Reasoned Action, whereby individual attitudes serve as the most proximal predictor of behaviour and are mediated solely by behavioural intention (Fishbein & Ajzen, 2010). Taken together, these findings on school operational culture add to literature documenting how addressing a school’s learning environment could offer an effective means of reducing levels of violence experienced by students (Freiberg, 1999; Gottfredson et al., 2005).

Outside of the school setting, differences were observed in normative beliefs in communities surrounding control and intervention schools, based on aggregated reports from caregivers at follow-up. Compared to communities surrounding control schools, communities surrounding intervention schools were less accepting of physical discipline practices both in school and at home. It may be that these changes in normative beliefs resulted from caregivers’ conversations with their children about the intervention taking place. Or perhaps the changes reflect caregivers’ awareness of a violence-reduction intervention taking place in their child’s school, even if the caregivers were themselves not personally involved in the intervention.

These findings on observed differences in normative beliefs over 18-months of the Good School Toolkit’s delivery are very promising, particularly given that the Toolkit is centred on the school rather than the home environment. Researchers and practitioners have highlighted the importance of addressing normative beliefs among parents in attempting to reduce violence against students, based on literature demonstrating links between social norms external to schools and the occurrence of violence within schools (Benbenishty, Zeira, & Astor, 2002; Kim et al., 2000). In this study, caregivers’ beliefs were thought to influence the Toolkit’s successful delivery in several ways. In a few instances, programme implementers reported that a parent decided to transfer their children out of an intervention school upon learning about the purpose of the Good School Toolkit; here, caregivers’ attitudes posed a threat to the acceptability of the violence-prevention intervention. Addressing caregivers’ beliefs was also deemed essential if the intervention effects observed on staff’s violence usage (Devries, Knight, et al., 2015) were to be sustained beyond the study timeframe. The study’s findings ultimately reinforce the Toolkit’s intentional engagement of caregivers in addition to staff and students.

Differences between control and intervention schools were not observed regarding the degree to which physical discipline is practiced within the home, highlighting potential value in engaging caregivers more directly in future Toolkit iterations. It is worth noting that the results were consistently in the direction of lower levels of violence at home in intervention versus control schools. The lack of significance may reflect a deficiency in power; although the Toolkit had a large effect in school, effects outside the school are likely to be smaller and would thus require a larger trial to detect. It may also be that fundamentally shifting disciplinary practices in the home would require continued effort beyond the 18-month timescale of the intervention’s delivery. While a greater percentage of caregivers reported violence in the home than students, these findings are consistent with other studies comparing parent and child reports of violence (Shahinfar, Fox, & Leavitt, 2000). The discrepancy may have been due to a smaller number of questions on specific acts of violence asked to the students compared with caregivers; the questionnaires for students centred primarily on acts of violence from school staff, the main trial’s primary outcome.

Although the intervention beyond the school setting did not appear to influence physical discipline practices within the home, findings on normative beliefs suggest potential for a school-based intervention to serve as a platform for wider change within communities. Other studies have shown that school-based health programmes—for instance, addressing physical activity in the United States (Berniell, De La Mata, & Valdes, 2013)—can influence the behaviours of parents at home. There is a need for a greater understanding of “spill-over” effects of school-based interventions into surrounding communities (de Heer, Koehly, Pederson, & Morera, 2011).

To our knowledge, this study is the first of its kind to address effects of an intervention for reducing staff violence on school operational culture in a low or middle-income setting. This study also explores intervention effects within communities surrounding schools, which represents an area of research that is rarely addressed. Additional strengths of this study include its large sample size, use of a cluster-randomised controlled trial design, high response rates among students and staff, and low levels of missing data.

Several study weaknesses should be noted, first, regarding the measures employed. Instruments to measure school operational culture and normative beliefs were developed for the purposes of this study, cognitively tested, and tested for face and construct validity and internal consistency. Full validation and reliability testing were beyond the scope of this study. Some variables using Likert or Likert-type response options were analysed as composite measures and others as single items. Though these measures were piloted, they may not have fully described the constructs under study. Future studies should conduct psychometric testing of measures to improve the interpretability and generalisability of the constructs. Additionally, in accordance with gold-standard practice, measures of violence against children in the home are self-reported. While interviewers were trained extensively in non-judgemental data collection techniques, some under-reporting is likely—though under-reporting is unlikely to fully account for the lack of significance for these measures.

Additionally, it was not possible to control for caregivers’ baseline characteristics given that caregiver data was only collected at follow-up. The 66% of caregivers who participated may have been more supportive of their child’s school environment or the Good School Toolkit, versus those who did not participate. Aggregated caregiver reports may not be fully representative of community-wide views; and future studies looking at spill-over effects of school-based interventions should invest in surveying wider samples of community members. Finally, as in other complex interventions, participants and data collectors were not masked, which may have biased the results towards larger effects but is unlikely to fully account for the differences observed.

In this study, schools were sampled to be representative of larger schools in the Luwero District, 100 percent of schools agreed to participate, and no school dropped out of the study. Follow-up response rates were high for students (92%) and staff (83%), and findings are thus broadly generalizable to Luwero District. Findings may also provide insight into school violence in other settings in Uganda and other sub-Saharan African countries with similar cultural norms and physical discipline practices. Given the 66% participation rate among caregivers and the inability to control for caregivers’ baseline characteristics, caution should be taken in generalising findings from caregiver surveys, including findings on normative beliefs, beyond Luwero District.

## 5 Conclusions

This secondary analysis of a cluster-randomised controlled trial sought to investigate effects inside and outside the school of a complex, school-wide intervention addressing staff violence against children in Luwero District, Uganda. Analyses centred on intervention effects on school operational culture, as well as normative beliefs about physical discipline practices and experiences of violence against children in the home. Results indicate that the Good School Toolkit achieved impacts on many components of school operational culture. Of note, the intervention also made inroads in transforming deeply-rooted norms around physical discipline practices outside of the school setting. These promising findings suggest that a school-based violence-prevention intervention can produce positive changes not only within the school itself but also within communities surrounding schools. Future research should investigate the Toolkit’s effects over long time periods and at greater scale.

## Funding

This work was funded by the MRC/DfID/Wellcome Trust via the Joint Global Health Trials Scheme and the Hewlett Foundation and the Oak Foundation.
